# Dilemmas and options for COVID-19 vaccination in children

**DOI:** 10.1186/s13052-023-01513-9

**Published:** 2023-08-25

**Authors:** Jingzhi Wen, Xiaoan Du, Adan Li, Shungeng Zhang, Shengyun Shen, Ziteng Zhang, Liyuan Yang, Changqing Sun, Jianing Li, Shiheng Zhu

**Affiliations:** 1 Department of Paediatrics, Yantai Yeda Hospital, Yantai, Shandong 264006 China; 2 Jining Medical University, Jining, Shandong 272067 China; 3 Cheeloo College of Medicine, Shandong University, Jinan, 250012 China

**Keywords:** COVID-19, Children, Vaccination, Omicron, Vaccine effectiveness, Bivalent vaccine

## Abstract

Over 16 million children have been detected positive for the coronavirus disease 2019 (COVID-19) in the United States since the outbreak of the pandemic. In general, children infected with severe acute respiratory syndrome coronavirus type 2 tend to have lighter symptoms than adults. However, in some cases, the infection can develop into severe forms, such as multisystem inflammatory syndrome in children. Moreover, long-term public health preventive interventions have had some negative effects on the physical and mental health of children. Given the important role that vaccination plays in reducing severe illness and mortality, it is essential for the efficient implementation of vaccination in the pediatric population. Nevertheless, parental distrust of vaccination, especially with regard to its safety and efficacy, hinders this process. Herein, we comprehensively summarize the available data on the safety and effectiveness of COVID-19 vaccine in children. The results show that the currently approved COVID-19 vaccine is safe and effective for children. Although two doses of vaccine in children seem insufficient to prevent Omicron infection, the booster dose provides enhanced protection against infection and severe illness. Most importantly, the bivalent vaccine has been approved for use in the pediatric population to extend the immune response to currently circulating Omicron variant. And the immune protection afforded to newborns after maternal vaccination appears to last only 6 months. Therefore, in the current situation where the rate of virus mutation is accelerating and the COVID-19 pandemic is still severe, it is crucial to extend vaccine protection to children over 6 months of age to weave a tighter safety net.

## Introduction

The coronavirus disease 2019 (COVID-19), caused by severe acute respiratory syndrome coronavirus 2 (SARS-CoV-2) infection, remains a major global healthy, social, and economic burden. It is characterized by the presence of respiratory disease with flu-like symptoms such as dry cough, body aches, and fever. In more severe cases, the disease can rapidly progress to pneumonia, acute respiratory syndrome, and even death [1]. As of June 7, 2023, there were more than 767 million confirmed cases worldwide, including more than 6.9 million deaths, and this number is rising sharply every day [2]. Moreover, SARS-CoV-2 causes acute respiratory infections of varying degrees of severity in different age groups. Older individuals (> 60 years) tend to have severe diseases and have the highest risk of death [3]. In contrast, most of the children with SARS-CoV-2 infection have a milder disease with common symptoms including fever, pharyngitis, cough, and diarrhea [4]. Still, in rare cases, children may also develop more serious consequences.

According to the latest data released by the Centers for Disease Control and Prevention, 17.2% of the nearly 100 million known cases of COVID-19 infection in the United States were in children [5]. Of the 16.3 million pediatric cases, 179,338 required hospitalization and intensive care [6]. Similar to adults, children who are obese, diabetic, or have immunocompromising conditions can increase the risk of admission to intensive care [7]. Although it is very rare, some previously healthy children may also experience severe diseases after the initial infection, such as multisystem inflammatory syndrome in children (MIS-C) [8]. Furthermore, considering the high proportion of children in COVID-19 cases, they may play an important role in the transmission of SARS-CoV-2, especially the highly transmissible Omicron variant [9, 10].

Although stringent public health interventions have been implemented to contain further transmission of SARS-CoV-2, including wearing facemasks, social distancing, and limiting social contact, the COVID-19 epidemic has not yet been fully controlled. Moreover, the above measures have created problems of educational disruption and social isolation, which may adversely affect the social and emotional development and mental health of children [11, 12]. It is also impossible for the public to comply with conservation measures for many years. Thus, there is a significant need to protect children through vaccination. In addition to the benefits to individuals, vaccination of children against COVID-19 can have beneficial knock-on effects in the community, thereby contributing to herd immunity in all age groups. But current data show that only 5.7% of children under four years old in Canada have received at least one dose of the vaccine [13]. Similarly, low vaccination rates were observed among children aged 2–4 and 5–11 years in the United States, with only 10.9% and 40.0% respectively [14]. Therefore, it is essential to summarize the available evidence and information to promote vaccination rates for children and to help achieve substantial control of community transmission in the future.

## Methods and materials

We reviewed the articles published on Google Scholar, PubMed, and Web of Science using the following terms: COVID-19, children, vaccination, Omicron, vaccine effectiveness, and bivalent vaccine. After an additional manual search and an extension of the search to the reference lists of all retrieved articles, 70 papers were finally included in our review. The types of studies included were prospective, retrospective, cross-sectional and case–control studies and one review. In addition, information from 14 official public health websites was also used.

### Factors affecting vaccine uptake in children

It is well known that widespread COVID-19 vaccination is the most effective and cost-effective method to interrupt SARS-CoV-2 transmission. However, the lower COVID-19 vaccination rates among children seem not to achieve substantial control of community transmission, which may prolong the outbreaks and epidemics of disease. For underage children, parents are usually the primary decision-makers on their children’s vaccination. Hence, we searched three databases to find the relevant literatures published since 2022 on the determinants of parents’ unwillingness to vaccinate their children with COVID-19 vaccine and made Table 1 [15–26].

**Table 1:** **Determinants of parental hesitancy to vaccinate their children against COVID-19**

Vaccine hesitancy determinants	Factors	Hesitancy (-) / Acceptance (+)	Place of study	Reference(s)
Individual/group determinants	**Beliefs, attitudes about health and prevention**			
	Belief that COVID-19 is not a serious illness	-	USA, Italy	[15, 16]
	Belief that children were not susceptible to infection	-	USA	[17]
	COVID-19 fear	+	Germany	[18]
	Acceptance and adherence to vaccination policies	+	Germany	[18]
	**Experience with past vaccination**			
	Parents’ past experiences of vaccination	+	USA, Germany	[17, 18]
	Children’s past experiences with vaccination	+	USA	[19]
	**Other factors**			
	A low proportion of COVID-19 among family members	-	China	[20]
**Contextual determinants**	**Perception of the pharmaceutical industry/government**			
	Distrust in government, science, or the pharmaceutical industry	-	USA, Germany	[17, 18]
	**Communication and media environment**			
	Obtain information about COVID-19 vaccine from the government and public health agencies	+	Thailand	[21]
	Conflicting media messaging	-	Italy, USA, China	[16, 17, 22]
	**Policies/politics**			
	Mandatory vaccination	-	Hong Kong (China)	[23]
	School resumption	+	Hong Kong (China)	[23]
	**Sociodemographic related**			
	Female	-	USA	[19, 24]
	Higher education level	-	Hong Kong (China), USA, China	[15, 20, 23]
		+	Italy, USA, Thailand, Japan	[16, 17, 21, 25]
	High income	-	China, Hong Kong (China)	[20, 23]
		+	USA, Japan	[17, 25]
	Lower socioeconomic status	-	USA, Thailand, Japan	[17, 21, 25]
	Married/cohabitant	+	Italy	[16]
	Parents older age	+	USA, Germany	[17, 18]
	Asian	+	USA	[17, 24]
	Black/Hispanic parents	-	USA	[15, 17, 19]
	Single child	+	Italy	[16]
	Children’s poor health	-	China	[20]
	Younger children	-	USA, China	[17, 22]
**Vaccine determinants**	**Risk/benefit (scientific evidence)**			
	Concern of efficacy of COVID-19 vaccines	-	Italy, USA, Thailand, Canada	[16, 17, 21, 26]
	Lack of long-term study results on vaccine efficacy	-	Thailand	[21]
	Vaccine no longer worked against new variants	-	USA, China	[17, 22]
	Concern of safety of COVID-19 vaccines	-	USA, Germany, Thailand, Canada	[15, 17, 18, 21, 26]
	Concern about vaccination side effects	-	USA, Thailand	[15, 21]
	**Cost**			
	Free vaccination	+	USA	[15]
	**Role of healthcare professional**			
	Having their child’s doctor who recommended the vaccine	+	Italy	[16]
	**Other factors**			
	Lack of community and family support for pediatric vaccination	-	USA	[17]
	Lack of choices of vaccine	-	Hong Kong (China)	[23]
	Rapid development of vaccine	-	Canada	[26]

Note: USA, United States of America.

Based on the result, parental decisions about whether to vaccinate their children are complex and multidimensional, including individual/group determinants, contextual determinants, and vaccine determinants (Table 1). In terms of the individual determinants, some parents still believe that the prevalence of COVID-19 infection in children is minimal and they are unlikely to progress to serious diseases after infection. However, as mentioned above, current data suggest otherwise. Moreover, studies have demonstrated that children whose parents have received or are willing to receive COVID-19 vaccine are more likely to receive the vaccine. It is worth mentioning that a study found that parental acceptance of the COVID-19 vaccination for children was closely related to the acceptance of previous influenza vaccination. Specifically, parents who refused to have their children vaccinated against influenza were more than five times as likely to refuse to have their children vaccinated against COVID-19 than other parents [27].

The most common factors that influence vaccine acceptance at the contextual level are gender, race, and educational level. According to Table 1, fathers are more likely to vaccinate their children than mothers in general. Considering that females tend to experience more adverse events after receiving COVID-19 vaccine, they may be more concerned about the potential side effects of COVID-19 vaccine on their children [28]. However, this result differs from a previous study in Poland by Babicki et al. (2021) in which they found that fathers were less likely than mothers to believe that vaccines can protect the health of children [29]. This may be due to differences in the social roles of fathers or mothers between communities. With respect to ethnicity, Blacks and Hispanics have shown an increased distrust of COVID-19 vaccination given the potential for discrimination, racism, and abuse within the healthcare system. Moreover, the effect of parental education level on vaccine hesitancy is a controversial issue. On the one hand, the higher the level of parental education, the more confidence they have in vaccination. On the other hand, highly educated parents may expect more information about vaccines before deciding to vaccinate their children, thereby leading to vaccine hesitancy. Notably, conflicting media messages, especially a lot of misinformation and anti-vaccine propaganda, may have a negative impact on the willingness to vaccinate during the pandemic.

Additionally, the younger age of children was also one of the reasons why parents refuse to vaccinate them. One cross-sectional study conducted in the United States showed that only 40% of parents were willing to vaccinate their children aged between 6 months and 4 years against COVID-19 [30]. A similar level of COVID-19 vaccination acceptability was found among parents with children under 18 months in the United Kingdom [31]. Currently, the safety and efficacy of the vaccine have become the most important considerations for parents who are hesitant or reluctant to vaccinate their children due to the rapid development and the lack of adequate information on the COVID-19 vaccine. Thus, there is an urgent need to comprehensively summarize and discuss the relevant information on the safety and effectiveness of the COVID-19 vaccine for children in order to improve vaccine acceptance and coverage.

### The safety and efficacy of COVID-19 vaccination among children

To date, the COVID-19 vaccine has shown favorable safety for children in most clinical studies [32, 33]. Although adverse events such as injection-site pain, fever, fatigue, and headache were reported after the vaccination in children, these symptoms were mild to moderate in severity and transient, resolving in a few days [34]. Moreover, there is no significant difference in the incidence of adverse reactions after vaccination in subjects aged 3–17 years and those aged 18–59 years or over 60 years or older [35, 36]. As a serious disease of cardiovascular system, myocarditis has been reported many times in pediatric patients with SARS-CoV-2 infection and has greatly increased the incidence rate and mortality of the SARS-CoV-2 in children [37, 38]. The appearance of myocarditis may be caused by an over-activation of the innate immune system and pro-inflammatory cytokine surge according to recent research [39]. Notably, the cases of myocarditis and pericarditis associated with the COVID-19 vaccine were also observed in the pediatric population, especially those following the mRNA vaccination. Moreover, this type of myocardial injury has typically presented in adolescent males and appears to be dose and interval dependent [40, 41]. Fortunately, these cases were of a mild and benign clinical course, and most patients recovered with only pain treatment. Although a few patients continued to have mild cardiac abnormalities during long-term follow-up, their incidence was significantly lower than after SARS-CoV-2 infection [42, 43].

As mentioned before, MIS-C is a hyper-inflammatory syndrome with multi-organ involvement caused by SARS-CoV-2 infection. There is evidence that children diagnosed with MIS-C have a robust and sustained immune response to SARS-CoV-2, including higher levels of spike protein and viral nucleocapsid protein antibodies [44]. Notably, if the COVID-19 vaccine can induce similar antibody responses in children as well, it may put otherwise healthy children at risk for serious outcomes following vaccination designed to prevent SARS-CoV-2 disease. Fortunately, hundreds of millions of children are vaccinated worldwide, but very few studies have reported vaccination as a potential trigger for the development of MIS-C in patients with no history of infection [45, 46]. Also, a national cohort study from France found a 94% reduction in the incidence of MIS-C among children aged 12–18 years after one dose of the BNT162b2 vaccine; no cases of MIS-C were reported among fully vaccinated children [47]. In line with this, Nygaard and colleagues (2022) found that the incidence of MIS-C was 11 per million vaccinated adolescents aged 5 to 17 years. In contrast, 183 cases of MIS-C were reported among one million SARS-CoV-2 infected individuals in the same age group [48]. The potential risk of vaccination on children’s health should be compared with the adverse consequences of COVID-19 rather than considered separately in view of the rapid and widespread spread of COVID-19.

Experiments on the effects of different COVID-19 vaccines have revealed that most COVID-19 vaccines were immunogenic and induced robust humoral responses in children [34, 49]. Moreover, there are non-inferior or even better immune responses in children and adolescents compared to that observed in adults. Concretely speaking, following two 3.0 µg doses of an inactivated SARS-CoV-2 vaccine (CoronaVac), the geometric mean titers (GMTs) of 142.2 in children 3–17 years old induced better immunogenicity than that elicited in adults aged 18–59 years (44.1) and those aged 60 years and older (42.2) with the same immunization schedule [32]. The neutralizing antibody response observed in aged 12–17 years after two doses of the mRNA vaccine was identical to that seen 18–24 years age group [50]. However, Xia et al. (2022) reported that seroconversion rate and neutralizing antibody titer in the 3–5 years age group are lower than those in adults aged 18–59 years on day 28 after one dose BBIBP vaccination [35]. Given those children aged 3–5 years may have less developed immune systems, this phenomenon was not surprising. It is universally acknowledged that the thymus gland of a young child is not fully developed. A thymic perivascular space constitutes the thymus, which contains granulocytes, mast cells, and other lymphoid cells and peaks in size between 10 and 25 years of age [51]. Additionally, young children have a reduced number and function of innate and adaptive immune cells and poorer production of immune media compared with adults [52]. It is worth mentioning that the serum conversion rates and neutralizing antibody titers in the age group of 3–5 years reached a level similar to that in the adult group after the second dose of the vaccine [35].

The intensity of the antibody response increased with increasing vaccine doses in all age groups of children (Table 2) [32–35, 49, 50, 53]. Specifically, from 3 to 17 years old, the neutralizing antibody titers induced by a 3.0 µg dose were higher than those of the 1.5 µg dose after the CoronaVac and the neutralizing antibody GMT of 2.0 µg BBIBP-CorV was significantly lower than that of the 4.0 and 8.0 µg [32, 35]. However, this does not mean that higher doses of vaccines should be offered to children, but rather that they should be considered in conjunction with potential adverse reactions caused by the vaccine. Moreover, the antibody titers induced by the same vaccine dose at different ages seem to be different (Table 2). In general, older children with more developed immune systems will produce higher neutralizing antibody titers. Nevertheless, the data in table two does not quite fit this pattern. In view of the small cumulative number of subjects, the outcome may have a certain partial bias. Hence, larger multicenter trials and sample analyses are required to produce convincing results.

**Table 2:** Immunogenicity of the COVID-19 vaccine in children

Type of vaccination	Age group	Number of subjects	Vaccination status	Dosage	Immunogenicity (95% CI)	Detection time interval	Reference
BBIBP-CorV	3–5 years	81	First dose	2.0 µg	GMT: 14.5	28 days	[35]
		83		4.0 µg	GMT: 20.2		
		82		8.0 µg	GMT: 21.3		
	6–12 years	84		2.0 µg	GMT: 30.0		
		84		4.0 µg	GMT: 54.1		
		83		8.0 µg	GMT: 55.8		
	13–17 years	83		2.0 µg	GMT: 48.0		
		83		4.0 µg	GMT: 61.4		
		82		8.0 µg	GMT: 80.2		
CoronaVac	3–5 years	46	Second dose	1.5 µg	GMT: 94.1	28 days	[32]
		45		3.0 µg	GMT: 140.5		
	6–11 years	69		1.5 µg	GMT: 90.3		
		68		3.0 µg	GMT: 139.7		
	12–17 years	71		1.5 µg	GMT: 78.3		
		67		3.0 µg	GMT: 146.0		
BNT162b2	5–11 years	264	Second dose	10.0 µg	GMT: 1197.6	1 month	[53]
	16–25 years	253		30.0 µg	GMT: 1146.5		
	12–15 years	190	Second dose	30.0 µg	GMNT_50_: 1239.5	1 month	[34]
	16–25 years	170			GMNT_50_: 705.1		
mRNA-1273	6–23 months	230	Second dose	25.0 µg	GMC: 1781	28 days	[49]
	2–5 years	264		25.0 µg	GMC: 1410		
	6–11 years	320		50.0 µg	GMPNAT: 1610		[33]
	12–17 years	340		100.0 µg	GMPNAT_50_: 1401.7		[50]

Note: GMT, geometric mean titer; GMNT_50_, geometric mean 50% neutralizing titer; GMC, geometric mean concentrations; GMPNAT, geometric mean pseudovirus neutralizing antibody titer; GMPNAT_50_, geometric mean 50% pseudovirus neutralizing antibody titer.

It is well known that compared to the original SRAS-CoV-2 strain, the Omicron variant has multiple mutations in the spike protein, especially in the receptor binding domain and the N-terminal domain, leading to extensive immune escape [54]. Hence, some parents are concerned that the COVID-19 vaccine, which was designed based on wild-type viral strains, may not provide adequate immune protection for children (Table 1). Indeed, the vaccine effectiveness (VE) against symptomatic infection was significantly lower during the Omicron-dominant period than during the pre-Omicron period for adolescents aged 12 to 18 years after two doses of the BNT162b2 vaccine (Table 3) [55–65]. The vaccination program for the 5–11 years age group was only approved late in 2021, so all the VE studies for this age group were conducted during the Omicron period. A study from Italy reported that the VE against infection in this age group declined rapidly to 21.2% within 3 months after full vaccination [61]. Although the third monovalent dose increased protection against symptomatic infection, the VE of monovalent booster doses against COVID-19 associated hospitalization has also inevitably declined with the passage of time [66]. Notably, mRNA-1273.214 vaccine prepared from Wuhan-Hu-1 and Omicron BA.1 spike protein mRNAs induced a strong neutralizing antibody response to Omicron BA.4 and BA.5 subvariants in adults, which is thought to play a constructive role in defense against the evolving virus [67, 68]. So far, several studies have shown that the bivalent vaccine is safe and reliable in all age groups of children [69, 70]. Therefore, the Centers for Disease Control and Prevention and the American College of Obstetricians and Gynecologists recommend that children receive an age-appropriate dose of a bivalent mRNA booster ≥ 2 months after completion of a COVID-19 primary series or receiving a monovalent booster dose to expand the immune response against the currently prevalent Omicron variant and to improve the protective effect of the COVID-19 vaccine against severe disease [71, 72].

**Table 3:** Vaccine effectiveness against omicron variant in children

Variant	Years	Vaccination status	Type of vaccination	Detection time interval	Vaccine effectiveness (95% CI)			Reference
					Symptomatic infection	Hospital admission	Admission ICU	
Omicron	5–11	First dose	BNT162b2	14–27 days	18.0%	NP		[55]
				≥ 14 days	9.0%			[56]
		Second dose		≥ 7 days	49.0%			
				14–30 days	60.1%			[57]
				30–90 days	28.9%			
				≥ 7 days	NP	82.7%	NP	[58]
				≥ 14 days		68.0%		[59]
				7–21 days	48.0%	NP		[55]
				51 days (median)	NP	48.0%	NP	[60]
				0–14 days	38.7%	NP		[61]
				43–84 days	21.2%			
Omicron	12–18	First dose	BNT162b2	122 days (median)	26.1%	NP		[62]
			CoronaVac	31 days (median)	21.5%			
		Second dose	BNT162b2	189 days (median)	54.9%			
			CoronaVac	64 days (median)	55.0%			
			BNT162b2	2–22 weeks	NP	43.0%	NP	[59]
				23–44 weeks		38.0%		
Pre-Omicron				2–22 weeks		93.0%		
				23–44 weeks		92.0%		
				7–21 days	93.0%	NP		[63]
				≥ 14 days	NP	94.0%	98.0%	[64]
				62 days (median)	93.0%	NP		[65]
Omicron		Third dose	BNT162b2	31 days (median)	86.8%	NP		[62]
			CoronaVac	67 days (median)	92.0%			

Note: ICU, intensive care unit; NP, not provided.

### Recommendations for the age range of childhood vaccinations

Epidemiological evidence suggests that infants born to mothers who received two doses of the mRNA vaccine during pregnancy had a 61% lower risk of hospitalization for SARS-CoV-2 in the first 6 months of life [73]. However, the levels of anti-spike IgG declined steadily after 6 months of life, and protection against SARS-CoV-2 infection was significantly diminished [74]. A study conducted in Israel observed a higher risk of infection among infants aged 0–1 year than among older children [75]. This result is understandable because the immune system of infants is not fully developed and unable to produce sufficient immunoglobulins [76]. Moreover, high levels of the SARS-CoV-2 virus in asymptomatic children may allow transmission of infection to family members who are in close contact with them [77]. Thus, it is essential to vaccinate children over the age of 6 months. Studies have demonstrated that participants aged 6–23 months have a strong immune response after three doses. Their GMT was similar to that observed in the group of people aged 16–25 years received two doses of BNT162b2 vaccine [78]. Additionally, the majority of adverse events reported in these trials were mild to moderate in severity, with no serious vaccine-related adverse events reported [78]. Currently, several international organizations have suggested BNT162b2 and mRNA-1273 vaccines for children over 6 months of age (Table 4) [79–84].

**Table 4:** Guidelines by various international organizations for COVID-19 vaccination in children

Organization	Type of vaccine	Developer(s)	Applicable age	Reference
WHO	BNT162b2 (mRNA vaccine)	BioNTech + Fosun Pharma + Pfizer	a. ≥ 12 years: SAGE recommends two doses (30 µg, 0.3 ml each), 4–8 weeks apart given intramuscularly. b. 5 to 11 years: recommends two doses (10 µg, 0.2 ml each) given intramuscularly and provided 4–8 weeks apart. c. 6 months to 4 years: three doses (3 µg, 0.2 ml each): a schedule of two doses 3 weeks apart followed by a third dose at least 8 weeks after the second dose.	[79]
	mRNA-1273 (mRNA vaccine)	Moderna + NIAID	a. 12 to 17 years: two doses (100 µg, 0.5 ml each), given intramuscularly, 4 weeks apart. b. 6 to 11 years: two doses (50 µg in 0.25 ml each), 4 weeks apart. c. 6 months to 5 years: two doses (25 µg, 0.25 ml each), 4 weeks apart.	[80]
FDA	BNT162b2 (mRNA vaccine)	BioNTech + Fosun Pharma + Pfizer	a. 6 months to 4 years: children who have received two doses of the monovalent Pfizer-BioNTech COVID-19 vaccine can receive the bivalent Pfizer-BioNTech COVID-19 vaccine as the third dose.	[81]
	mRNA-1273 (mRNA vaccine)	Moderna + NIAID	a. 6 months to 5 years: children can receive the bivalent Moderna COVID-19 vaccine as a booster dose at least 2 months after completion of primary vaccination with the monovalent Moderna COVID-19 vaccine.	
CDC	Novavax (Protein subunit vaccine)	Novavax, Inc.	a. 12 to 17 years: Novavax is not authorized as a booster dose at this time and can only be used for primary series.	[82]
	BNT162b2 (mRNA vaccine)	BioNTech + Fosun Pharma + Pfizer	a. ≥ 12 years: recommends three doses, a schedule of two doses 3–8 weeks apart followed by a third dose at least 2 months after the second dose. b. 5 to 11 years: recommends three doses given intramuscularly and provided 3–8 weeks apart, children aged 5 years can only get a Pfizer-BioNTech booster, and children aged 6–11 years can get a Pfizer-BioNTech or Moderna booster at least 2 months after second dose or last booster. c. 6 months to 4 years: recommends three doses, a schedule of two doses 3 weeks apart followed by a third dose at least 8 weeks after the second dose.	
	mRNA-1273 (mRNA vaccine)	Moderna + NIAID	a. 6 years above: a schedule of two doses, 4–8 weeks apart followed by a third dose at least 2 months after second primary series dose. b. 6 months to 5 years: 3 doses, 4 to 8 weeks apart. At least 2 months after second Moderna dose, children aged 6 months–4 years can only get a Moderna booster, and children aged 5 years can get a Pfizer-BioNTech or Moderna updated booster.	
EMA	Comirnaty (mRNA vaccine)	BioNTech + Fosun Pharma + Pfizer	a. ≥ 12 years: two doses of 30 micrograms each, 3 weeks apart. b. 5 to 11 years: two doses of 10 micrograms each, 3 weeks apart. c. 6 months to 4 years: three doses of 3 micrograms each, the first two doses are given three weeks apart, followed by a third dose given at least 8 weeks after the second dose.	[83]
	Spikevax (mRNA vaccine)	Moderna + NIAID	a. ≥ 12 years: two doses of 100 micrograms each, 28 days apart. b. 6 to 11 years: two doses of 50 micrograms each, 28 days apart. c. 6 months to 5 years: two doses of 25 micrograms each, 28 days apart.	[84]

Note: WHO, World Health Organization; FDA, Food and Drug Administration; CDC, Centers for Disease Control and Prevention; EMA, European Medicines Agency; NIAID, National Institute for Allergy and Infectious Diseases; SAGE, Strategic Advisory Group of Experts on Immunization.

## Conclusion

Children, as a special population, need to consider many influencing factors when receiving the COVID-19 vaccine. Among these, the most important considerations are the safety and efficacy of the vaccine. While there are some common adverse reactions to vaccination in children, these events are mild and transient, and disappear within a few days. Compared to adults, there was no significant difference in the incidence of adverse effects after vaccination in children. Although cases of vaccine-associated myocarditis have been identified in the pediatric population, such sequelae are rare and affected patients experience rapid resolution of symptoms and recovery of cardiac function in a short period of time. Additionally, vaccination of children not only induces a strong immune response, but also provides protection against symptomatic diseases, especially in preventing hospitalization. Notably, the protection afforded to children after two doses of vaccine appears to be relatively limited due to extensive immune escape of the omicron variant. The protection provided by three doses of monovalent vaccine still decreases rapidly over time. Fortunately, bivalent vaccines have been approved for use in children and may provide broader and longer-lasting immune protection.

The level of SARS-CoV-2 antibody provided by the mother significantly decreased after 6 months of birth. Given the high risk of infection and the high transmissibility of children after infection, it is essential to vaccinate children over 6 months of age in a timely manner. Only in this way can establish a stronger immune barrier as early as possible to deal with the constant mutation of the virus.

## Data Availability

Not applicable.

## Abbreviations

COVID-19: Coronavirus disease 2019
GMTs: Geometric mean titers
MIS-C: Multisystem inflammatory syndrome in children
SARS-CoV-2: Severe acute respiratory syndrome coronavirus 2
VE: Vaccine effectiveness
